# Implementing real-time immunometabolic assays and immune cell profiling to evaluate systemic immune response variations to *Eimeria* challenge in three novel layer genetic lines

**DOI:** 10.3389/fvets.2023.1179198

**Published:** 2023-04-18

**Authors:** Krysten Fries-Craft, Susan J. Lamont, Elizabeth A. Bobeck

**Affiliations:** Department of Animal Science, Iowa State University, Ames, IA, United States

**Keywords:** immunometabolism, Fayoumi, Leghorn, flow cytometry, *Eimeria* spp.

## Abstract

**Introduction:**

Evaluating differences in immune responses to *Eimeria spp*. between poultry genetic lines could be valuable for understanding favorable traits to address coccidiosis, a costly poultry disease. The objective was to compare peripheral blood mononuclear cell (PBMC) immunometabolism and composition during *Eimeria* challenge in three distinct and highly inbred genetic lines; Leghorn Ghs6, Leghorn Ghs13, and Fayoumi M5.1.

**Methods:**

At hatch, 180 chicks (60/ line) were placed in wire-floor cages (10 chicks/cage) and fed a commercial diet. Baseline PBMC were isolated on d21 (10 chicks/line) and 25 chicks/line were inoculated with 10X Merck CocciVac®-B52 (Kenilworth, NJ), creating 6 genetic line × *Eimeria* groups total. Chicks were euthanized on 1, 3, 7, and 10d post-inoculation (pi; 5 chicks/ line × *Eimeria* group) for PBMC isolation with body weight and feed intake recorded throughout. Immunometabolic assays to determine PBMC ATP production profiles and glycolytic activity were implemented along with flow cytometric immune cell profiling. Genetic line × *Eimeria* challenge, and line´challenge fixed effects were analyzed using the MIXED procedure (SAS 9.4; *P* ≤ 0.05).

**Results and Discussion:**

Before inoculation, M5.1 chicks had 14.4-25.4% greater average daily gain (ADG) with 19.0-63.6% increased monocyte/macrophage^+^, Bu-1^+^ B cell, and CD3^+^ T cell populations compared to both Ghs lines (*P* < 0.0001) but similar immunometabolic phenotype. The *Eimeria* main effect reduced ADG by 61.3% from 3–7dpi (*P* = 0.009) except in M5.1 chicks, where no ADG difference due to challenge was found. At 3dpi, *Eimeria*-challenged M5.1 chicks had 28.9 and 33.2% reduced PBMC CD3^+^ T cells and CD3^+^CD8α^+^ cytotoxic T cells than unchallenged chicks, suggesting early and preferential recruitment from systemic circulation to tissues local to *Eimeria* challenge (i.e., intestine; *P* ≤ 0.01). Both Ghs lines displayed 46.4–49.8% T cell reductions at 10dpi with 16.5–58.9% recruitment favoring underlying CD3^+^CD4^+^ helper T cells. Immunometabolic responses in *Eimeria*-challenged Ghs6 and Ghs13 chicks were characterized by a 24.0–31.8% greater proportion of ATP from glycolysis compared to unchallenged counterparts at 10dpi (*P* = 0.04). These results suggest that variable T cell subtype recruitment timelines in addition to altered systemic immunometabolic requirements may work synergistically to determine favorable immune responses to *Eimeria* challenge.

## 1. Introduction

Enteric diseases such as coccidiosis caused by parasitic *Eimeria spp.* present significant challenges to the poultry industry. Over its life cycle, *Eimeria* causes significant damage to the chicken intestine resulting in depressed feed intake (FI), reduced intestinal integrity, and nutrient malabsorption, ultimately contributing to significant reductions in production performance (1, 2). Severity ranges from feed efficiency-reducing subclinical infections to severe clinical disease with mucoid or hemorrhagic diarrhea and high mortality (3). Economic losses due to coccidiosis have been estimated as high as $14.5 billion (USD) annually in both broiler and layer production with subclinical infections being cited as significant contributors to these losses (4, 5). While prevention and treatment strategies are available, vaccination against *Eimeria* is not cross-protective between the different species known to infect birds. Resistance to anticoccidial drugs is ubiquitous and their use is not allowed in antibiotic-free or organic production systems (6, 7). Collectively, this illustrates a significant problem wherein *Eimeria* infections are poorly controlled and economically damaging even when not considered clinically severe.

Improving bird immunity against *Eimeria* is a principal goal identified by many researchers but relies on understanding underlying immune processes to pinpoint potential biological targets. One strategy to accomplish this is to compare *Eimeria* challenge immunological outcomes between susceptible and resistant bird genetic lines. Such comparisons can be made between Leghorn and Fayoumi genetic lines identified as being susceptible and resistant, respectively, to pathogens including avian influenza virus, Newcastle disease virus (NDV), and *Salmonella* Enteritidis (8–10). The Leghorn breed as the foundation stock for many commercial layer genetic lines has a history of artificial selection for productivity traits, sometimes at the expense of disease resistance, whereas the Fayoumi breed as a predominantly backyard bird and genetically distant from commercial lines has been subjected to natural selection in favor of disease resistance traits (11, 12). Within the Leghorn breed, Ghs6 and Ghs13 lines are congenic for the major histocompatibility complex (MHC), a genomic region with many immune-modulating genes, on the same genetic background (13). Comparative outcomes during NDV challenge have categorized Leghorn Ghs6 and Ghs13 lines as “low” and “high” responders, respectively, in regard to immune-related gene expression within the lung; however, how these lines compare during intestinal *Eimeria* infection has not been evaluated (14). Instead, genetic line comparisons during *Eimeria maxima* challenge have been made between Fayoumi M5.1 and M15.2 genetic lines. Previous work has identified Fayoumi M5.1 birds as being less susceptible to *Eimeria maxima* challenge based on reduced oocyst shedding, increased body weight (BW) gain, and increased jejunal pro-inflammatory interferon (IFN)-γ in favor of anti-inflammatory interleukin (IL)-10 expression (15). The Fayoumi M5.1 line was used in the current study for comparison of an established *Eimeria* “resistant” genetic line to unknown responders in Leghorn Ghs6 and Ghs13 lines. All three lines used in this study are highly inbred over 100 generations since 1954 (13).

In poultry, evaluating immune responses is complicated by the lack of available reagents; therefore, assessments based on gene expression are heavily featured in published literature. These studies have provided valuable insights into host responses to *Eimeria*, with particular emphasis on cytokine expression profiles within intestinal tissues (16–18). While valuable in terms of identifying timelines of cytokine response, these studies do not provide insight into systemic immunometabolic shifts that may be underlying production losses. During an immune response, quiescent immune cells favoring oxidative metabolism are activated and differentiate into effector cells in a process requiring rapid, but significantly less efficient, ATP production from glycolysis even in the presence of oxygen (19, 20). As a result of increased demand, inflammatory immune responses divert available energy away from animal growth, which has deleterious effects on developing chicks and further contributes to *Eimeria*-induced performance losses (21, 22).

Real-time cell culture-based immunometabolic assessment of chicken peripheral blood mononuclear cells (PBMC) has been optimized in previous experiments and provides specific *ex vivo* insights into cellular metabolic preferences during disease challenge allowing researchers to pinpoint when costly metabolic shifts might occur (23). As a heterogenous population of immune cells including leukocytes, monocytes, thrombocytes, and erythrocytes (24), combining real-time metabolic assays with immune profiling by flow cytometry allows for the potential identification of cell populations responsible for observed metabolic shifts. This has been done previously in assessing hen responses to *Staphylococcus aureus* but has not been similarly applied to *Eimeria* infection or genetic line comparisons of *Eimeria* immune responses (25). The study objective was to evaluate immunometabolic shifts and underlying cell profiles in chicken PBMC isolated from legacy genetic lines identified as being resistant (Fayoumi M5.1) and susceptible (Leghorn Ghs6 and Ghs13) during *Eimeria* challenge.

## 2. Materials and methods

### 2.1. Birds and housing

All protocols were approved by the Iowa State University Institutional Animal Care and Use Committee (protocol #19-156). At hatch, 180 chicks from Leghorn (Ghs6 and Ghs13) and Fayoumi (M5.1) genetic lines (60 birds/ line) were placed in 18 raised-wire floor cages within a single brooder unit (10 chicks/cage, Petersime Model 2SD20RE; Gettysburg, OH). Chicks were allowed *ad libitum* access to Purina® Start & Grow® non-medicated crumble diet (Purina Mills, LLC, Gray Summit, MO) and water. On d21, 10 chicks/genetic line were CO_2_-anesthetized for baseline blood collection by cardiac puncture before euthanasia by cervical dislocation. Half the remainder were randomly assigned to unchallenged or *Eimeria-*challenged groups. Unchallenged chicks were sham inoculated with phosphate-buffered saline (PBS) while challenged chicks were orally gavaged with 10X Coccivac®-B52 (Merck, Kenilworth, NJ) resulting in 6 total genetic line × *Eimeria* challenge groups. Additional blood samples were collected as previously described at 1, 3, 7, and 10 days post-inoculation (pi) from 5 chicks/group. Cage BW and FI were recorded on every sample collection day to calculate average daily gain (ADG) and average daily feed intake (ADFI). The duodenal loop, distal jejunum around Meckel’s diverticulum, and ceca from chicks euthanized for blood sampling were lesion scored at 7 and 10 dpi using scoring guidelines published by Johnson et al. (26). According to this system, a score of 0 indicates no observable lesions while disease severity increases from 1 to 5. Excreta were collected from manure pans under each cage and pooled by each genetic line × *Eimeria* challenge group to enumerate shed oocysts by McMaster chamber (FEC Source, Grand Ronde, OR) (27). Briefly, 2 g of pooled excreta were homogenized in 28 mL 1.2–1.25 specific gravity sucrose float solution, oocysts were visualized and enumerated using a microscope at 10X objective, and the sum of counted oocysts in each chamber grid was multiplied by 50 to determine oocysts/g of excreta. The study concluded on 10 dpi.

### 2.2. Real-time immunometabolic assays

Blood was collected by cardiac puncture under CO_2_ anesthesia into heparinized needles, syringes, and collection tubes before euthanasia by cervical dislocation. Methods adapted from Sandford et al. (28) were used to isolate PBMC. Briefly, 3 mL of both Histopaque 1119 and 1077 (Sigma Aldrich, St. Louis, MO) were carefully layered into a 15 mL centrifuge tube and blood diluted 1:1 in sterile PBS (approximately 6 mL total) was carefully pipetted onto the gradient. Following centrifugation at 650 ×*g* for 35 min (low acceleration and no brakes), cells were harvested from the 1077-PBS density gradient interface and washed twice in sterile PBS. No cells were visually apparent at the 1119-1077 interface and not collected from this density gradient. Isolated cells were enumerated by hemocytometer and plated in triplicate in a 96-well cell culture plate (200,000 cells/well) for Agilent Real-Time ATP and Glycolytic Rate Assays (Agilent, Santa Clara, CA). Remaining PBMC not used for immunometabolic assays were frozen in RPMI with 42.5% heat-inactivated chicken serum and 7.5% DMSO at −80°C using protocols and recommendations adapted from mammalian PBMC research (29).

All immunometabolic reagents were prepared according to manufacturer’s instructions and assays were run using the Seahorse XFe96 Analyzer (Agilent, Santa Clara, CA) at 40°C. The real-time ATP rate assay injects oxidative metabolic inhibitors oligomycin (15 μM) followed by rotenone + antimycin A (Rot/AA; 5 μM) into the cell culture media to systematically determine mitochondrial and glycolytic contributions to ATP production. The glycolytic rate assay specifically tests glycolytic pathways by injecting 5 μM Rot/AA to inhibit oxidative metabolism followed by 500 mM 2-deoxy-D-glucose (2-DG) to inhibit glycolysis. Unlike the real-time ATP rate assay, the glycolytic rate assay specifically measures glycolytic shifts following Rot/AA injection to determine the cellular ability to meet energy requirements through glycolysis (compensatory glycolysis) and residual glycolysis not fully inhibited by 2-DG (post-2-DG acidification). Measures taken in the real-time ATP and glycolytic rate assays are converted to ATP production rate (pmol ATP/min) and proton efflux rate (PER; pmol/min), respectively, by Wave Software (Agilent, version 2.6.1) and exported for data analysis.

### 2.3. Peripheral blood immune cell profiles

Frozen PBMC were thawed, washed in PBS, enumerated, and aliquoted into polystyrene tubes prior to analysis. The analysis panel consisted of innate immune cell and lymphocyte markers: monocyte/macrophage biotin (clone KUL01; mouse IgG_1_κ), Bu-1 Alexa Fluor® 647 (clone AV20; mouse IgG_1_κ), cluster of differentiation (CD)3 Alexa Fluor® 700 (clone CT-3; mouse IgG_1_κ), CD4 PE-Cy7 (clone CT-4; mouse IgG_1_κ), CD8α Pacific Blue™ (clone CT-8; mouse IgG_1_κ), and T cell receptor (TCR) γδ PE (clone TCR-1; mouse IgG_1_κ) all antibodies and associated isotype controls were from Southern Biotech (Birmingham, AL). Each PBMC sample was stained with fluorescence-minus-one and associated isotype controls to account for non-specific binding. Briefly, cells in the first tube of the set were stained with all the extracellular markers combined in a single mix. In subsequent tubes, a single marker was systematically removed from the mix and replaced with its corresponding isotype control.

Following primary antibody addition, cells were incubated in the dark for 30 min at 4°C. To label biotin-conjugated primary antibodies, cells were washed in PBS and BV785-conjugated streptavidin (BioLegend, San Diego, CA) diluted in PBS was added to each flow cytometry tube. After incubating in the dark for 30 min at 4°C, cells were washed and resuspended in PBS before being analyzed by BD FACSCanto™ cytometer (BD Biosciences, San Jose, CA). Cell population data were analyzed by FlowJo software (BD Biosciences, version 10.5.0). Within FlowJo, cells were gated according to forward scatter properties (area vs. height) to identify singlets and live cells were defined as cell populations with non-zero forward and side scatter within singlet populations. Cells positive for each marker were determined visually by areas of cell separation from the overall population within the data file and gate placement was confirmed by the signal disappearance and reappearance between tubes with or without the marker of interest. Monocyte/macrophage^+^, Bu-1^+^, and CD3^+^ were identified within singlet-live cell populations whereas CD4^+^, CD8α^+^, and TCRγδ^+^ cells were identified within CD3^+^ populations.

### 2.4. Statistical analysis

Bird BW, ADG, and ADFI data were analyzed using the following statistical model:


$$y_{ijk}=\mu +L_{i}+E_{j}+{\left(L\times E\right)}_{ij}+BW_{i}+e_{ijk}$$

where y_ijk_ is observed effect, μ is the overall mean, L_i_ is the genetic line main effect at the ith level (*i* = 3; Ghs6, Ghs 13, or M5.1), E_j_ is the *Eimeria* challenge main effect at the jth level (*j* = 2; ± *Eimeria*), (L × E)*_ij_* is the genetic line and *Eimeria* challenge interaction, BW_i_ is the initial BW covariate, and e*_ijk_* is the associated error. This same model was used for lesion score, immunometabolism and cell population data without the initial BW covariate. Analysis using these models was completed using the MIXED procedure (SAS 9.4) with significance denoted at *p* ≤ 0.05. The percent difference between significantly different means was calculated to quantify magnitude of the observed changes in this study.

## 3. Results

### 3.1. Body weight and feed intake

M5.1 chicks were consistently the heaviest, weighing 7.3% and 9.1% more than Ghs6 and Ghs13 chicks, respectively, at hatch and 12.1% and 21.1% more on d21 (*p* < 0.0001; Table 1). While both Leghorn genetic lines had similar BW at hatch, after 21d, Ghs6 chicks weighed 9.9% more than Ghs13 chicks (*p* < 0.0001). Prior to inoculation, M5.1 chicks had a 14.4% and 25.4% greater ADG compared to Ghs6 and Ghs13 chicks, respectively (*p* < 0.0001) but had an intermediate ADFI between Ghs6 and Ghs13 lines (Table 1). Within the Leghorn genetic lines in this study, Ghs6 had a 12.8% and 24.8% higher ADG and ADFI, respectively, compared to Ghs13 chicks prior to inoculation (*p* ≤ 0.01).

**Table 1:** Body weight and feed intake measures among Leghorn (Ghs6 and Ghs13) and Fayoumi (M5.1) inbred genetic lines before and after inoculation with 10X Coccivac-B52 (Merck Animal Health, Kenilworth, NJ).

	Treatment						SEM	*P-*values^1^		
Measure	Ghs6		Ghs13		M5.1			Line	*Eimeria*	Line × *Eimeria*
day 0 BW, g^2^	24.83^b^		25.33^b^		27.33^a^		0.28	< 0.0001	N/A^3^	N/A
day 21 (baseline) BW, g	127.5^b^		114.9^c^		145.7^a^		6.00	< 0.0001	N/A	N/A
day 0–21 ADG, g	4.86^b^		4.24^c^		5.68^a^		0.29	< 0.0001	N/A	N/A
day 0–21 ADFI, g	23.45^a^		17.64^b^		19.24^ab^		3.88	0.01	N/A	N/A
	**Ghs6**	**Ghs6 + *Eimeria***	**Ghs13**	**Ghs13 + *Eimeria***	**M5.1**	**M5.1 + *Eimeria***				
1 dpi BW, g	131.50	139.90	119.60	120.30	153.20	156.50	10.44	<0.0001	0.13	0.47
Baseline-1 dpi ADG, g	5.67	11.00	4.00	6.00	8.33	10.67	4.29	0.03	0.009	0.40
Baseline-1 dpi ADFI, g	20.44	21.31	15.19	15.85	25.98	24.56	4.63	0.0008	0.97	0.71
										
3 dpi BW, g	141.70	149.90	130.60	131.30	169.90	174.90	23.12	0.01	0.42	0.86
1–3 dpi ADG, g	5.03	4.93	5.32	5.32	8.59	9.14	9.65	0.87	0.93	0.99
1–3 dpi ADFI, g	32.17	34.83	25.50	20.83	28.17	34.50	12.43	0.04	0.63	0.35
										
7 dpi BW, g	188.90	171.40	158.60	126.60	187.80	193.80	43.16	0.01	0.18	0.38
3–7 ADG, g	11.80	5.39	6.98	−1.18	4.47	4.79	6.29	0.04	0.009	0.12
3–7ADFI, g	31.98	23.60	20.56	13.23	19.94	23.69	11.67	0.03	0.18	0.25
										
10 dpi BW, g	171.90	186.00	168.70	139.70	245.30	245.80	63.37	0.05	0.75	0.5
7–10 ADG, g	5.78	4.78	3.33	4.22	19.33	17.44	13.84	0.29	0.35	0.36
7–10 ADFI, g	32.33	42.00	21.33	25.00	23.00	37.00	14.33	0.04	0.03	0.56

1 Data represent the mean measurement (6 cages/genetic line before inoculation; 3 cages/treatment after inoculation) on a per bird basis. Values with different letter superscripts are significantly different, *P* ≤ 0.05.

2 BW, body weight; ADG, average daily gain; ADFI, average daily feed intake.

3 Effects pertaining to Eimeria inoculation were not included in the model for pre-inoculation measures.

Genetic line effects on BW and ADFI remained consistent throughout the post-inoculation period with M5.1 chicks being 22.5%–25.3% heavier than Ghs13 chicks but having an intermediate ADFI while Ghs6 chicks consumed 25.6%–39.2% more feed than Ghs13 chicks (*p* ≤ 0.05; Table 1). *Eimeria* inoculation reduced ADG from 3 to 7 dpi by 61.3% in challenged vs. unchallenged birds, regardless of genetic line (*p* = 0.009). The significance of this main effect was largely attributed to reduced ADG observed in both Ghs lines while ADG was numerically similar between unchallenged and challenged M5.1 birds from 3 to 7 dpi (Table 1). From 7 to 10 dpi, ADG was similar between challenged and unchallenged chicks, but the *Eimeria* main effect increased ADFI 26.3% in challenged vs. unchallenged chicks (*p* = 0.03). The magnitude of increased ADFI was numerically greater in challenged vs. unchallenged Ghs6 and M5.1 genetic lines, suggesting that recovered or preserved ADG may be a function of compensatory feed intake in these chicks (Table 1).

*Eimeria-*inoculated chicks developed observable intestinal lesions by 7 and 10 dpi (*Eimeria* main effect; *p* ≤ 0.003; Table 2). The average duodenal lesion score in *Eimeria*-challenged Ghs6 chicks was 1.0 and 0.8 points lower than scores observed in *Eimeria-*challenged Ghs13 and M5.1 chicks, respectively (*p* = 0.02); however, by 10 dpi duodenal lesion scores were similar across *Eimeria*-challenged chicks in all genetic lines (Table 2). No lesion score differences in the jejunum and cecum were observed at 7 and 10 dpi in *Eimeria*-challenged birds from any genetic line. Infrequent oocysts were detected in the pooled excreta samples from unchallenged birds (Table 2).

**Table 2:** Average intestinal lesion scores and oocyst counts in Leghorn (Ghs6 and Ghs13) and Fayoumi (M5.1) inbred genetic lines ±10X Coccivac-B52 (Merck Animal Health, Kenilworth, NJ).

Sample	Treatment						SEM	*P*-values		
	Ghs6	Ghs6 + *Eimeria*	Ghs13	Ghs13 + *Eimeria*	M5.1	M5.1 + *Eimeria*		Line	*Eimeria*	Line × *Eimeria*
**7 dpi**										
Duodenum^1^	0^b^	0.2^b^	0^b^	1.2^a^	0^b^	1^a^	0.24	0.02	< 0.0001	0.02
Jejunum	0	0.2	0	0.6	0	0.8	0.28	0.33	0.003	0.33
Ceca	0	0.4	0	1	0	0.6	0.27	0.30	0.0003	0.30
Excreta^2^, oocysts/g	650	118,200	16,900	428,300	50	228,600	N/A^3^	N/A	N/A	N/A
**10 dpi**										
Duodenum	0	1.2	0	0.6	0	1.4	0.23	0.06	<0.0001	0.06
Jejunum	0	1	0	1	0	1.2	0.12	0.38	<0.0001	0.38
Ceca	0	1	0	1.2	0	1.4	0.26	0.56	<0.0001	0.56
Excreta, oocysts/g	2,200	14,000	3,200	12,650	1,700	14,250	N/A	N/A	N/A	N/A

1 Lesion scores done by one observer on 5 intestinal sections per treatment in accordance with the scoring system published by Johnson and Reid. 0 = no observed signs of disease. Averages with different letter superscripts are significantly different, *P* ≤ 0.05.

2 Oocysts were enumerated by McMaster chambers. Two gram of pooled excreta were mixed into 28 mL of 1.2–1.25 specific gravity float solution and enumerated under a microscope. Counts were multiplied by 50 to determine number of oocysts/g of excreta.

3 Sample pooled by treatment and could not be analyzed statistically.

### 3.2. Peripheral blood mononuclear cell ATP production

**Figure 1:**
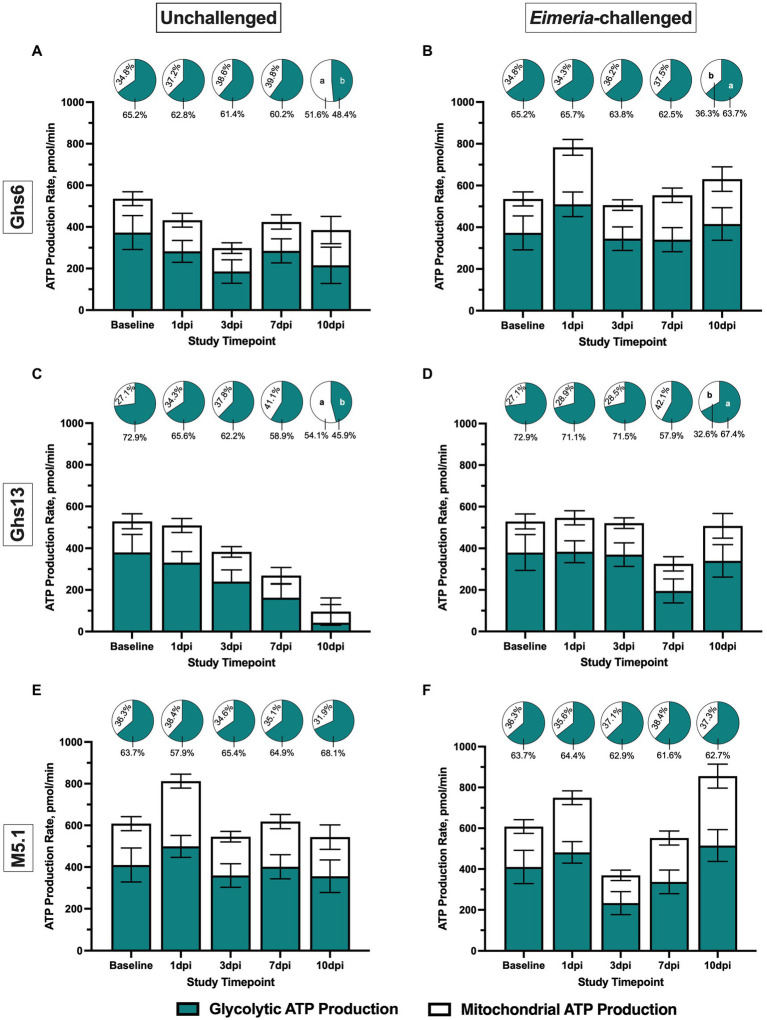
Peripheral blood mononuclear cell ATP production (200,000 cells/well) among Leghorn **(A,B)** Ghs6 and **(C,D)** Ghs13 and **(E,F)** Fayoumi M5.1 inbred genetic lines before and after inoculation with 10X Coccivac-B52 (Merck Animal Health, Kenilworth, NJ) as determined by the Agilent Real-Time ATP Rate Assay and Seahorse XFe96 analyzer (Santa Clara, CA). Data represent mean ATP production rate (pmol/min) attributed to glycolytic and mitochondrial metabolism (*n* = 5 birds/treatment) ± SEM. Filled and empty bars denote separate ATP contributions from glycolytic and mitochondrial metabolism, respectively, while the stacked bar (filed + empty) represents total ATP production. Pie charts above each bar denote the relative contribution of each pathway as a percentage of total ATP production. Different letter labels on same-colored slices within the same timepoint are significantly different, *p* ≤ 0.05.

Figure 1 illustrates glycolytic and mitochondrial contributions to ATP production over time in the various study groups with pie charts over each bar detailing the relative contribution of each pathway to overall ATP production. On day 21 (baseline), total ATP production and underlying contributions from glycolysis and mitochondrial respiration were similar between genetic lines prior to *Eimeria* inoculation (Figure 1). At 1 dpi, glycolytic and mitochondrial ATP production in M5.1 PBMC was 19.2% and 41.2% greater, respectively, compared to Ghs13 PBMC, culminating in 22.1% greater total ATP production without affecting the relative contributions of glycolysis and mitochondrial activity to the overall ATP production profile, regardless of *Eimeria* status (*p* ≤ 0.05; Figures 1C–F). This observed genetic line main effect was due to generally greater ATP production from glycolysis and mitochondrial respiration in both unchallenged and challenged M5.1 PBMC from baseline to 1 dpi, causing an *Eimeria*-independent 22.1% increase in total ATP production rate (Figures 1E,F). At 10 dpi, the *Eimeria* main effect contributed to greater glycolytic and mitochondrial ATP production by 51.7% and 48.6%, respectively, culminating in 48.6% greater total ATP production in challenged vs. unchallenged chicks (*p* ≤ 0.05; Figure 1). In *Eimeria*-challenged Ghs6 and Ghs13 chicks, the relative contribution of glycolysis to ATP production profiles was 24.0% and 31.8% greater, respectively, compared to corresponding unchallenged birds, suggesting metabolic shifts favoring glycolysis over oxidative phosphorylation in later stages post-inoculation (*p* = 0.04; Figures 1A–D).

### 3.3. Peripheral blood mononuclear cell glycolytic rate

**Figure 2:**
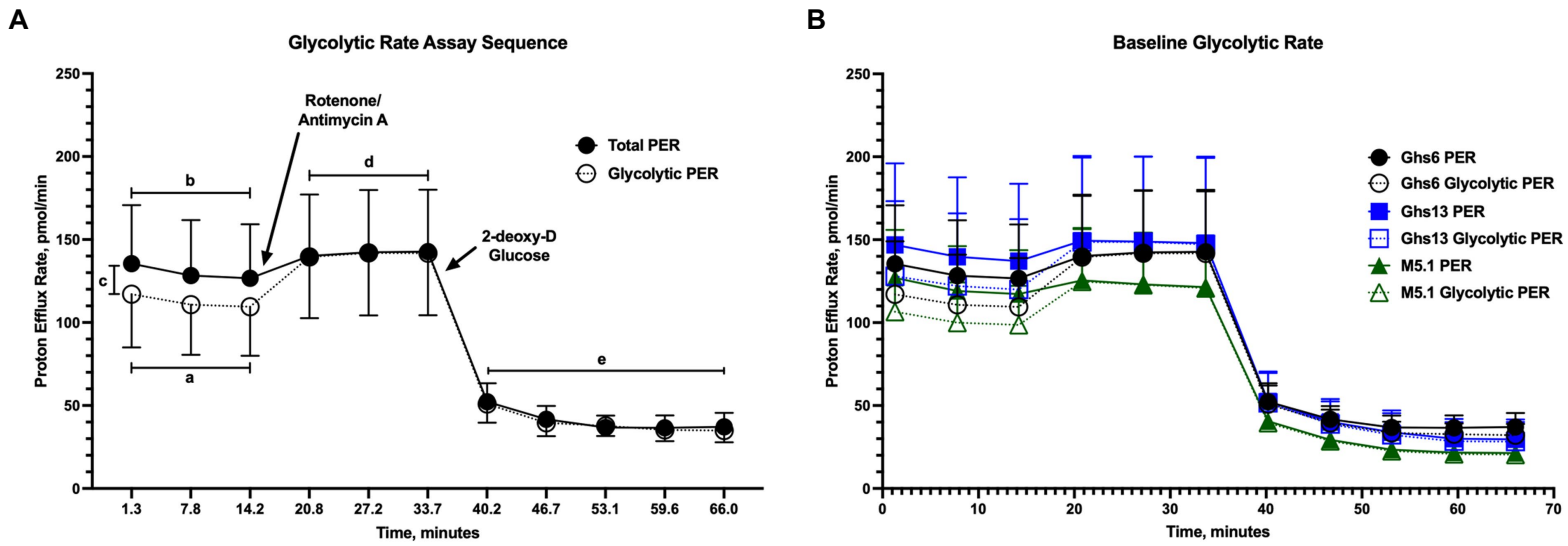
**(A)** Example output for the Agilent Glycolytic Rate Assay run on the Seahorse XFe96 Analyzer (Santa Clara, CA). The assay collects measurements to determine (a) basal glycolysis and (b) basal proton efflux rate (PER) prior to inhibition of mitochondrial oxidative phosphorylation by rotenone/antimycin A injection into assay media. In these basal measurements, (c) mitochondrial activity is determined as the difference between the total PER and glycolytic PER lines. Following inhibition of mitochondrial contributions to PER, (d) compensatory glycolysis—the ability of cells to utilize glycolysis to meet metabolic needs—is measured before injection of 2-deoxy-D glucose (2DG) into the assay media. Following glycolytic inhibition by 2DG, the assay measures (e) post-2DG acidification as a measurement of residual glycolytic activity. **(B)** Baseline glycolytic rate assay output in peripheral blood mononuclear cells isolated from 21 day-old Leghorn (Ghs6 and Ghs13) and Fayoumi (M5.1) inbred genetic lines before inoculation with 10X Coccivac-B52 (Merck Animal Health, Kenilworth, NJ) Data represent the mean proton efflux measurement (PER; *n* = 10 birds/genetic line) ± SEM.

**Figure 3:**
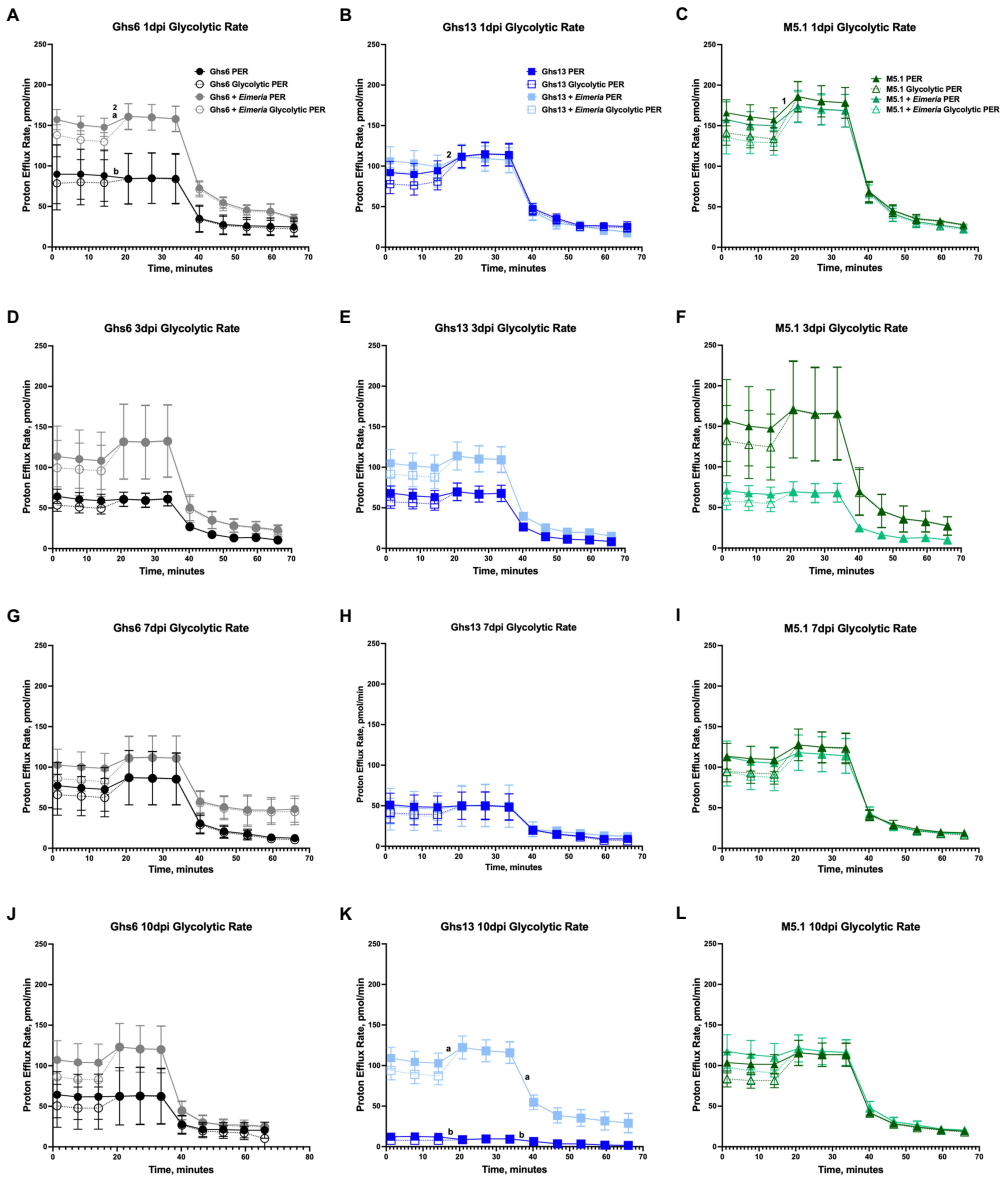
Glycolytic rate assay outputs in peripheral blood mononuclear cells isolated from Leghorn **(A,D,G,J)** Ghs6 and **(B,E,H,K)** Ghs13 and **(C,F,I,L)** Fayoumi M5.1 inbred genetic lines at **(A–C)** 1, **(D–F)** 3, **(G–I)** 7, and **(J–L)** 10 day post-inoculation (pi) with 10X Coccivac-B52 (Merck Animal Health, Kenilworth, NJ) as determined by the Agilent Glycolytic Rate Assay and Seahorse XFe96 analyzer (Santa Clara, CA). Data represent the mean proton efflux measurement (PER; *n* = 5 birds/treatment) ± SEM. Slopes between panels within the same timepoint labeled with a different number indicate a significant genetic line main effect while slopes with different letter labels within the same panel and timepoint denote a significant line **×** *Eimeria* effect (*p* ≤ 0.05).

Figure 2A shows assay outputs provided by the Seahorse XFe96 analyzer during the glycolytic rate assay and highlights regions corresponding to basal glycolysis, basal PER, compensatory glycolysis, and post-2DG acidification. No differences in pre-inoculation glycolytic rate were observed between genetic lines as shown by the similar assay outputs in Figure 2B. In these outputs, a flat PER line between measures 3 and 4 corresponding to Rot/AA injection suggests that cells increased glycolysis to meet, but not exceed, basal metabolic activity. This was generally observed in PBMC from unchallenged Ghs lines throughout the study (Figure 3). Supplementary Figure 1 presents the average of measurements taken within key regions of the assay for direct comparison.

When examining individual glycolytic rate outputs over the course of the study, several patterns emerged. Throughout the study, outcomes in Ghs13 chicks were obscured by progressively diminishing glycolytic rate outputs in unchallenged chicks over the course of the study (Figures 3B,E,H,K). At 1 dpi, distinct separation between unchallenged and challenged Ghs6 PBMC glycolytic rate outputs were observed and characterized by a positive PER slope between assay measures 3 and 4 in *Eimeria*-challenged Ghs6 PBMC compared to a relatively unchanged PER slope in their unchallenged counterparts. Underlying this change was an 83.9% increased glycolytic PER slope in challenged vs. unchallenged Ghs6 PBMC at 1 dpi (*p* = 0.01), suggesting an early glycolytic response to *Eimeria* challenge unique to the Ghs6 line (Figure 3A). Despite the apparent separation in challenged vs. unchallenged outputs throughout the study, average basal glycolysis, compensatory glycolysis, and post-2DG acidification between PBMC from unchallenged and *Eimeria*-challenged Ghs6 were increased numerically, but not statistically (Supplementary Figure 1).

At 1 dpi, the genetic line main effect contributed to a 41.6% and 81.0% increased PER and 43.0% and 52.5% increased glycolytic PER slope between measures 3 and 4 in M5.1 vs. Ghs6 and Ghs13 chicks, respectively, suggesting that M5.1 PBMC robustly mobilized glycolytic pathways during the assay to exceed basal metabolic activity (Figures 3A–C). Unchallenged and challenged glycolytic rates in M5.1 PBMC were generally similar over the course of the post-challenge period, except for 3 dpi when glycolytic rate was depressed in *Eimeria*-challenged M5.1 PBMC compared to unchallenged counterparts (Figure 3F). When key regions within the assay were averaged, *Eimeria*-challenged M5.1 chicks had 56.0% lower basal glycolysis, 55.2% lower basal PER, and 59.2% lower compensatory glycolysis compared to unchallenged M5.1 birds, indicating significant separation in assay outputs at 3 dpi (*p* ≤ 0.05; Supplementary Figure 1). Glycolytic rate in M5.1 PBMC recovered to outputs similar to unchallenged counterparts by 7 dpi (Figure 3I).

### 3.4. Peripheral blood immune cell populations

**Figure 4:**
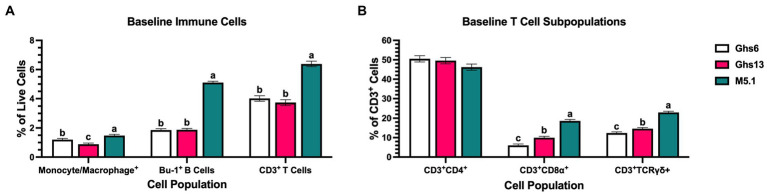
Baseline immune cell proportions as a percentage of peripheral blood mononuclear cells in Leghorn (Ghs6 and Ghs13) and Fayoumi (M5.1) inbred genetic lines before inoculation with 10X Coccivac-B52 (Merck Animal Health, Kenilworth, NJ). Analyzed immune cells comprise **(A)** monocyte/macrophage, Bu-1^+^ B, and CD3^+^ T cells in addition to **(B)** underlying CD4^+^ helper, CD8α^+^ cytotoxic, and TCRγδ^+^ T cell subpopulations. Data represent the mean population of cells positive for each marker within the **(A)** live cell or **(B)** CD3^+^ cell gates (*n* = 10 birds/genetic line) ± SEM. Bars with different letter labels are significantly different, *p* ≤ 0.05.

Evaluating immune population shifts within PBMC may identify which cells are contributing to observed metabolic patterns. Analyzed immune cell populations consisted of monocyte/macrophages as early innate immune responders and Bu-1^+^ B and CD3^+^ T cells as lymphocyte representatives of adaptive immunity. Within CD3^+^ T cell populations, subpopulations of CD3^+^CD4^+^ helper T (T_H_) cells, CD3^+^CD8α^+^ cytotoxic T (T_C_) cells, and CD3^+^TCRγδ^+^ (γδ) T cells were also assessed. Prior to inoculation, 21 day-old M5.1 chicks had 19.0% and 40.3% more peripheral monocytes/macrophages, 63.2% and 63.6% more Bu-1^+^ B cells, and 37.1% and 41.5% more CD3^+^ T cells than Ghs6 and Ghs13 chicks, respectively (*p* < 0.0001; Figure 4A). Underlying elevated T cell populations, M5.1 birds also had 46.6% and 67.7% more T_C_ and 36.4% and 46.1% more γδ T cells (*p* < 0.0001) without affecting T_H_ populations (Figure 4B).

**Figure 5:**
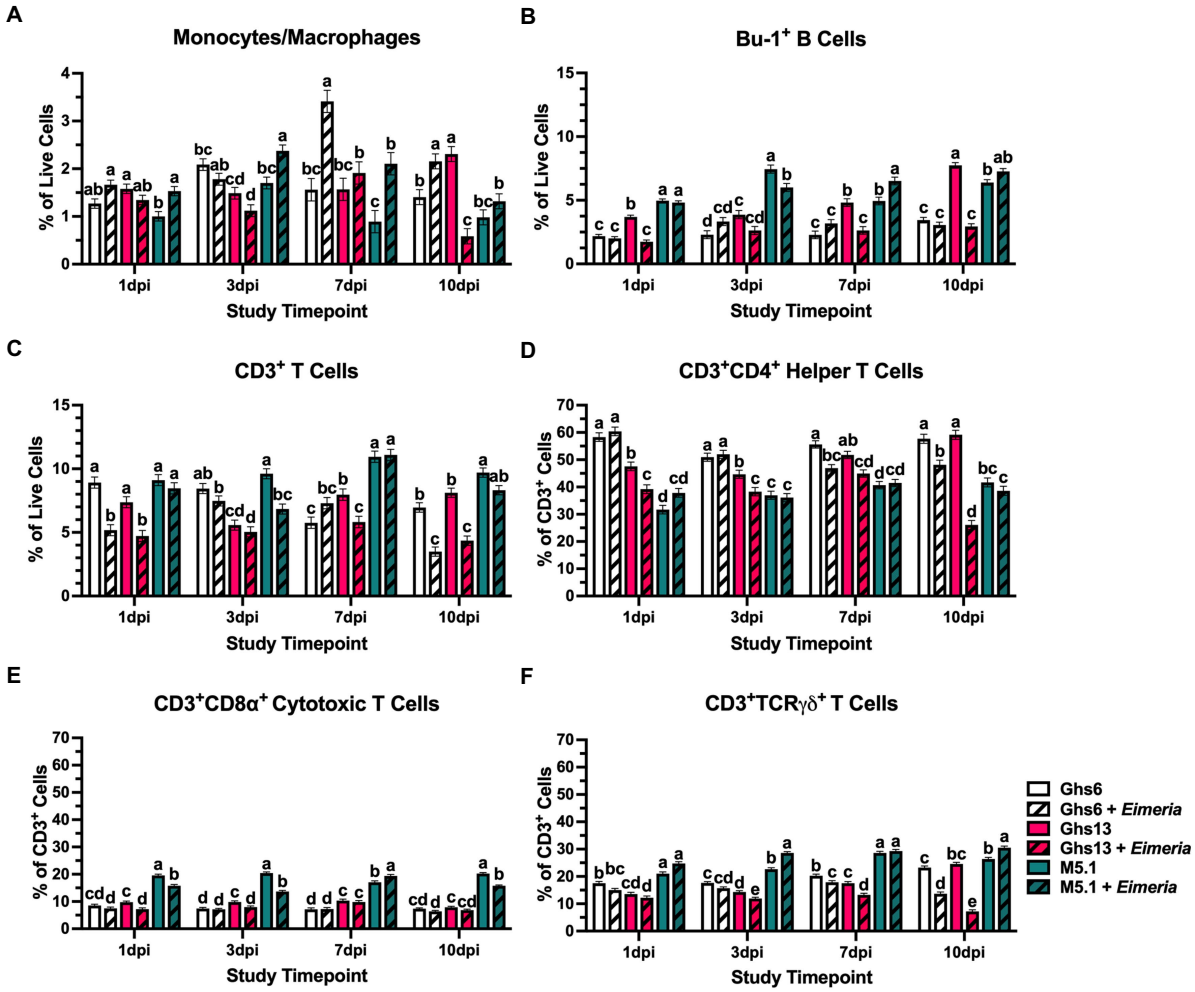
Percentages of **(A)** monocyte/macrophage^+^, **(B)** Bu-1^+^ B cells, **(C)** CD3^+^ T cells, **(D)** CD3^+^CD4^+^ helper T cells, **(E)** CD3^+^CD8α^+^ cytotoxic T cells, and **(F)** CD3^+^TCRγδ^+^ T cells in peripheral blood mononuclear cells isolated from Leghorn (Ghs6 and Ghs13) and Fayoumi (M5.1) inbred genetic lines at 1, 3, 7, and 10 days post-inoculation (pi) with 10X Coccivac-B52 (Merck Animal Health, Kenilworth, NJ). Data represent the mean percentage of cells positive for each marker among **(A–C)** live cells or **(D–F)** CD3^+^ cells (*n* = 5 birds/treatment) ± SEM. Within a post-inoculation timepoint bars with different letter labels are significantly different, *p* ≤ 0.05.

Over the course of the post-challenge period, the genetic line × *Eimeria* interaction was significant for every immune cell population at every timepoint analyzed, allowing for comparison of different immune cell response timelines (Figure 5). In *Eimeria*-challenged M5.1 chicks only, monocytes/macrophages were 34.5% higher than their unchallenged counterparts at 1 dpi (*p* = 0.003). Monocytes/macrophages remained elevated 36.0% and 57.7% in challenged vs. unchallenged M5.1 chicks at 3 and 7 dpi, respectively (*p* ≤ 0.006) but showed similar monocyte/macrophage^+^ cell percentages at 10 dpi indicating a sustained monocyte/macrophage response that recovered within 10 dpi. In a relatively delayed response, PBMC monocytes/macrophages were 54.3% higher in challenged vs. unchallenged Ghs6 chicks at 7 dpi and remained 34.8% elevated at 10 dpi (*p* ≤ 0.006). In contrast, monocyte/macrophage differences in *Eimeria*-challenged vs. unchallenged Ghs13 chicks were characterized by 74.7% lower populations at 10 dpi (*p* = 0.006), suggesting late recruitment to peripheral tissues without detectable prior expansion (Figure 5A).

As part of the adaptive immune system, B cell populations were expected to respond at later post-inoculation timepoints. At 1 dpi, only *Eimeria-*challenged Ghs13 chicks had 52.8% fewer Bu-1^+^ B cells compared to their unchallenged counterparts (*p* < 0.0001; Figure 4B). For the remainder of the study, peripheral Bu-1^+^ B cell populations in *Eimeria*-challenged Ghs13 chicks remained low, but stable, while increasing over time in unchallenged Ghs13, culminating in 45.4% and 62.0% greater populations in unchallenged vs. challenged Ghs13 at 7 and 10 dpi, respectively (*p* < 0.0001). Populations of B cells in *Eimeria*-challenged M5.1 chicks remained relatively stable from 3 to 7 dpi but fluctuated in their unchallenged counterparts resulting in the observed population differences within this genetic line. In contrast, B cell populations in Ghs6 chicks remained relatively stable over the course of the challenge period, regardless of challenge status (Figure 5B).

In addition to B cells, CD3^+^ T cells are important participants in the adaptive immune response and exhibit a variety of different effector functions. When examining overall CD3^+^ T cells, populations in unchallenged Ghs6 and Ghs13 generally increased from baseline to 1 dpi resulting in *Eimeria*-challenged Ghs6 and Ghs13 chicks having 41.9% and 36.0% fewer CD3^+^ T cells, respectively, compared to their unchallenged counterparts (*p =* 0.002; Figure 5C). At 3 dpi, peripheral CD3^+^ T cells in *Eimeria*-challenged M5.1 chicks were 28.9% lower compared to their unchallenged counterparts, suggesting that these cells were recruited early to peripheral tissues (*p* = 0.01). T cell populations in *Eimeria*-challenged M5.1 chicks recovered by 7 dpi. In contrast, 27.0% reductions indicative of recruitment were not observed in *Eimeria*-challenged Ghs13 chicks until 7 dpi (*p* = 0.0002) and 49.8% reductions in challenged Ghs6 chicks were not observed until 10 dpi (*p* = 0.002; Figure 5C).

Underlying these T cell population changes were differential shifts in the various effector T cell subpopulations. At 3 dpi, when there was evidence of early CD3^+^ T cell recruitment to peripheral tissues, *Eimeria*-challenged M5.1 chicks had 33.2% fewer CD3^+^CD8α^+^ T_C_ cells with 20.7% greater CD3^+^TCRγδ^+^ T cells compared to their unchallenged counterparts (*p* < 0.0001; Figures 5E, F). In Ghs13 chicks at 7 dpi when their corresponding CD3^+^ T cell recruitment occurred, *Eimeria*-challenged Ghs13 had 13.1% and 24.3% fewer CD3^+^CD4^+^ T_H_ and γδ T cell cells, respectively, compared to their unchallenged counterparts (*p* ≤ 0.001; Figures 5D,F). At 10 dpi, T_H_ and γδ T cell populations remained 58.9% and 70.8% reduced between challenged and unchallenged Ghs13 chicks, while *Eimeria*-challenged Ghs6 birds also had 16.5% and 41.1% reduced T_H_ and γδ T cell populations, respectively, compared to unchallenged counterparts (*p* < 0.0001; Figures 5D,F). Overall, these outcomes suggest that not only do the different genetic lines have differential T cell response timelines, but the recruited subpopulations vary between Leghorn and Fayoumi lines.

## 4. Discussion

Outcomes in this study can be summarized in two statements: (1) Fayoumi M5.1 birds generally displayed fewer negative effects on 3–7 dpi ADG and ADFI during *Eimeria* infection than their Leghorn Ghs6 and Ghs13 counterparts and (2) variations in the immune response between genetic lines could be linked to differential immune cell recruitment and immunometabolic kinetics. Support for the first statement is apparent when examining BW outcomes where M5.1 chicks maintained consistent BW and ADG between challenged and unchallenged groups throughout the study—a finding that was consistent with previous work by Kim et al. (15). Starch is a critical energy source and highly digestible in poultry (30). While comparative digestibility research in these genetic lines has not been conducted, outcomes from broiler research suggest that selection for increased production traits may negatively impact starch digestibility over time (31). As the Leghorn genetic lines represent those that have undergone selection for productivity, this may partially explain why Fayoumi M5.1 chicks weighed consistently more and were better able to maintain BW and ADG during *Eimeria* challenge. Additionally, *Eimeria* infection is associated with depressed FI (2), a finding that was numerically observed in both Leghorn lines from 3 to 7 dpi but not apparent in Fayoumi M5.1 birds during the study (Table 1). In contrast, both Ghs6 and Ghs13 chicks showed BW, ADG, and ADFI reductions between unchallenged and challenged groups around 7 dpi (Table 1). In Ghs6 chicks, the numerically depressed ADG from 3 to 7 dpi was largely due to ADG fluctuation in their unchallenged counterparts while the magnitude of ADG loss was more apparent in *Eimeria-*challenged Ghs13 chicks with negative ADG. These outcomes in the Leghorn genetic lines suggest that Ghs6 chicks may be less susceptible to *Eimeria*-induced performance reductions than Ghs13 chicks, but further research is needed.

The 10X dose of vaccine-strain *Eimeria* used in this study consisted of four different species: *E. acervulina, E. maxima, E. tenella,* and *E. mivati*. Each species infects different intestinal sections with *E. acervulina, E. mivati,* and *E*. *maxima* targeting the duodenum and/or jejunum and *E. tenella* infecting the ceca (2, 32). Studies using vaccine-strain *Eimeria* typically induce a subclinical infection and the low average lesion scores in this study (<2) are consistent with other findings using high-dose CocciVac®-B52 (33, 34). Housing chicks in raised wire-floor cages reduced interaction of birds with their excreta and resultant *Eimeria* cycling to induce a synchronous challenge. As such, lesion scoring and oocyst shedding changes from 7 to 10 dpi more likely indicate variations over the course of a single cycle (Table 2). Lesion scores generally increased from 7 to 10 dpi in all tissues; however, the meaning of this is uncertain because it could not be linked to simultaneous performance changes and there is no consensus regarding the connection between gross lesion scores and animal health (35, 36) (Tables 1, 2).

Oocyst detection in unchallenged birds despite no observable intestinal lesions may be explained by the housing system used (Table 2). Manure pans in this study collected excreta from 2 side-by-side cages and *Eimeria*-challenged cages were randomly distributed throughout the brooder. It is possible that in the process of removing pans, excreta were dislodged from the wire floors or inadvertently mixed between challenged and unchallenged cages, confounding measurement of oocyst shedding outcomes.

In addition to BW and ADG, differences in underlying immune responses to *Eimeria* could potentially explain variations in disease resistance and identify alterations that protect bird health. Fayoumi M5.1 birds had consistently greater populations of monocytes/macrophages, Bu-1^+^ B cells, CD3^+^ T cells, and a shift in underlying T cell populations that favored T_C_ and γδ T cells compared to both Leghorn lines (Figure 4). It is important to note that many γδ T cells in chickens are also CD8^+^ and there may be potential overlap within these cell populations (37, 38). Despite this overlap, T_C_ and γδ T cells are both associated with cytotoxic effector functions in chickens with mammalian research indicating that the TCRγδ structure may enable stronger proliferative signals and faster onset of effector functions (38, 39). While the exact function of these T cell subsets in chickens is not clear and further functional research is needed, baseline expansion of γδ T cells and other immune cell populations in Fayoumi M5.1 PBMC suggests their immune systems may be primed to quickly respond to intracellular pathogens like *Eimeria* without altering immunometabolic profiles or negatively impacting growth.

During the post-challenge period, the immunometabolic profiles of Ghs13 chicks were characterized by a decline in both ATP production and glycolytic rate over time in unchallenged chicks while their *Eimeria*-challenged counterparts maintained relatively consistent immunometabolic profiles (Figures 1, 3). While overall ATP production and glycolytic rate declined in unchallenged Ghs13 birds, underlying metabolic pathways shifted to favor ATP production by oxidative phosphorylation over glycolysis (Figure 1C). In contrast, underlying metabolic profiles in *Eimeria*-challenged Ghs13 favored glycolytic over oxidative metabolism (Figure 1D). Compared to glycolysis, oxidative phosphorylation is more energetically efficient, producing 36 ATP molecules per glucose molecule compared to just 2 ATP produced by glycolysis (19). Immunometabolic reductions over time in unchallenged Ghs13 could be due to age, but a 5-fold reduction over 10 days is drastic and future research is needed to better elucidate age-related changes in PBMC immunometabolism between unchallenged genetic lines. Regardless, maintained energy production in the PBMC of *Eimeria*-challenged Ghs13 birds by means of less-efficient pathways associated with inflammatory signals suggests a sustained, energetically costly, systemic response that may be underlying observed negative effects on BW and ADG (Table 1).

Despite being from the same breed, immunometabolic patterns in Ghs6 birds differ from those observed in their Ghs13 counterparts. In unchallenged Ghs6 chicks, the immunometabolic response to *Eimeria* was characterized by a quick 31.5% increase in total ATP production from baseline to 1 dpi that resolved to baseline levels by 3 dpi (Figure 1B). At the same time, all glycolytic rate measures in challenged vs. unchallenged Ghs6 birds were numerically greater, suggesting that not only were PBMC producing more ATP in response to *Eimeria* but were more glycolytically active at a baseline state within the glycolytic rate assay and better at utilizing glycolysis to meet energy demands during in-assay metabolic inhibition (Figure 3). Increased glycolysis and glycolytic rate following *Eimeria* challenge could indicate quick immune activation as this metabolic pathway provides rapid energy to support the demands of proliferating and differentiating immune cells (20). Like their Ghs6 counterparts, 10 dpi relative glycolytic contributions to overall ATP production were increased in *Eimeria*-challenged Ghs6 lines due to a sudden shift toward oxidative metabolism from 7 to 10 dpi in unchallenged Ghs6 birds. The reason for this shift is biologically uncertain and obscures *Eimeria*-induced metabolic changes while further emphasizing the need for further research to evaluate age-related immunometabolic shifts in these genetic lines.

While the early systemic immunometabolic response in *Eimeria-*challenged Ghs6 birds was intriguing, no significant shifts in underlying PBMC populations were observed in challenged vs. unchallenged Ghs6 birds at 1 dpi (Figure 5). This could indicate that glycolytic immune activation signals were detected without corresponding proliferation, potentially elevated by PBMC thrombocyte and erythrocyte presence (40), or that immune cell populations more susceptible to damage during cryopreservation or not analyzed in the current panel, like dendritic cells (DC), may be responsible for this metabolic shift. In addition to antigen presentation, avian DC activate innate immunity in the early responses to pathogen challenge (41). As such, increased DC populations could potentially explain the observed ATP increase at 1 dpi in *Eimeria*-challenged Ghs6 birds, but no specific DC markers are available. Previous research has estimated their presence in chicken PBMC by co-expression of CD11c, MHC-II, and CD45 (42). These markers are present on a multitude of other cell types and need to be analyzed in the same panel to specifically identify avian DC (43–45). Similarly, erythrocyte and thrombocyte presence could be accounted for flow cytometrically *via* K55 or CD45 antibodies to evaluate these as potential metabolic contributors (40); however, it is not certain how significantly these cells contribute to immunometabolic differences during pathogen challenge since their presence would be expected in PBMC isolated from all animals. With the limited number of commercially available poultry immunological reagents and few fluorophore options for flow cytometry, this means that DC detection or thrombocyte/ erythrocyte elimination comes at the expense of analyzing other cell populations.

Three notable features were observed in the immunometabolic responses of Fayoumi M5.1 chicks. First, both *Eimeria* challenged and unchallenged chicks showed 18.8%–25.1% numerically increased total ATP production from baseline to 1 dpi that resolved to baseline in unchallenged chicks by 3 dpi (Figures 1E,F). Due to the quick resolution and short time period between baseline and 1 dpi sample collections, there could be acute stress from handling birds over 2 consecutive days (although unhandled controls were not included), or age-related changes. This indicates that stress could be a confounding factor when using metabolic assays in challenge studies that require repeated handling over a short time interval to track the immune response. Identifying specific stress-related signatures in immunometabolic outcomes and evaluating differences between poultry genetic lines could optimize the use of immunometabolic assays and produce specific recommendations for their implementation in pathogen challenge studies.

Second, the biggest deviation in ATP production and glycolytic rate occurred at 3 dpi when these measures were decreased in *Eimeria*-challenged vs. unchallenged M5.1 birds (Figures 1, 3; Supplemental Figure 1). As increased metabolic activity and glycolysis are hallmarks of immune activation, this significant reduction was unexpected but corresponded with the simultaneous 28.9% reduction in PBMC T cells in *Eimeria*-challenged M5.1 chicks compared to their unchallenged counterparts (Figure 5C). Such reductions from systemic circulation could indicate immune cell recruitment to infected tissues such as the intestine to aid local immune responses. As T cells display a multitude of effector functions important to adaptive immunity and are expected to be recruited in the immune response during later stages around 7 dpi, this early shift could indicate a faster adaptive immune response in M5.1 birds during *Eimeria* infection. Additionally, since this recruitment occurred with simultaneous immunometabolic reductions it is possible that recruited T cells were activated systemically and prepared to respond quickly once recruited to the intestine.

Compared to mammals, birds naturally maintain higher homeostatic plasma glucose concentrations above 200 mg/dL, which may provide more available glucose for peripheral immune activation (46). This suggests that selection for improved production traits could result in age-related starch digestibility reductions that may alter the glucose environment for circulating immune cells. In turn, insufficient activation prior to recruitment from the peripheral blood could hinder immune responsiveness and worsen disease outcomes. While this is an intriguing possibility, further research is required to better elucidate connections between starch digestibility, plasma glucose, immune cell function, and disease outcomes in laying hens. It is also important to note that T cell populations, ATP production, and glycolytic rate all recovered in *Eimeria*-challenged M5.1 birds by 7 dpi, further supporting a connection between this specific immune cell population and immunometabolic outcomes (Figures 1, 3, and 5).

The expected or delayed T cell response in Ghs13 and Ghs6 lines, respectively, could have been detrimental to outcomes in Leghorn birds that could also be explained by differences in recruited T cell subpopulations as well. When overall T cells were potentially recruited from systemic circulation, simultaneous reductions in T_C_ cells were observed in *Eimeria*-challenged M5.1 birds whereas reduced T_H_ and γδ T cells were observed in both Ghs lines. While both T_H_ and T_C_ cells are important for anti-*Eimeria* immunity, T_C_ cells may produce IFN-γ, a cytokine associated with favorable BW outcomes when administered to *Eimeria*-infected chickens and identified as potentially underlying outcomes in early M5.1 *Eimeria* resistance studies (15, 47–49). This suggests that not only do *Eimeria*-challenged M5.1 birds have faster induction and resolution of T cell responses than Ghs6 and Ghs13 birds, but preferentially recruited T cell subtypes may be more effective at targeting *Eimeria* in a manner that preserves performance.

Third, overall ATP production by PBMC was increased in *Eimeria*-challenged M5.1 at 10 dpi without changes in glycolytic rate that would indicate immune cell activation (Figures 2F, 3L). Notably, oxidative phosphorylation contributions to ATP production were numerically increased 44.9% in *Eimeria*-challenged vs. unchallenged M5.1 birds. Oxidative metabolism—in addition to being more energetically efficient—is associated with regulatory immune cell activity and could indicate the beginning of immune response resolution not observed in the Leghorn genetic lines (50). Additionally, 10 dpi populations of potentially immunoregulatory γδ T cells were increased in *Eimeria*-challenged M5.1 birds compared to their unchallenged counterparts (51) (Figure 5F); however, immune cell profiling does not provide functional insights, and further research is needed.

Collectively, the results herein demonstrate multifactorial complexities in anti-*Eimeria* immune responses that may work synergistically and contribute to differential disease susceptibility between genetic lines. The assays used allowed for more comprehensive understanding of host immune responses to *Eimeria* than has previously been possible. In the comparatively more resistant Fayoumi M5.1 birds, *Eimeria* responses were characterized by primed baseline immunity, faster recruitment of T_C_ cells that may be more effective against *Eimeria,* with a detectable immunometabolic shift toward potential immune resolution by study end. Within the Leghorn Ghs lines, Ghs13 immunometabolic outcomes were characterized by sustained but not increased immunometabolism with expected T cell recruitment timelines that favored potentially less effective T cell subtypes. As an intermediate, Ghs6 birds were potentially less susceptible to performance losses than their Ghs13 counterparts but not as resilient as Fayoumi M5.1 birds with responses characterized by an early 1 dpi increase in ATP production and faster glycolytic activation but delayed T cell recruitment also favoring potentially less effective T cell subtypes. Although many of the key interpretations herein are based on numeric rather than statistically significant differences, they provide important foundational work for investigating *Eimeria* resistance traits. Future research using more replicates and applying cell sorting techniques prior to immunometabolic analysis could confirm initial observations while specific investigation into local intestinal *Eimeria* responses could potentially confirm the fate of recruited immune cells identified in this study.

## Data availability statement

The raw data supporting the conclusions of this article will be made available by the authors, without undue reservation.

## Ethics statement

The animal study was reviewed and approved by the Iowa State University Institutional Animal Care and Use Committee.

## Author contributions

EB and KF-C designed the study and chicks from each genetic line were provided by SL. KF-C conducted the study, collected samples, performed sample/statistical analysis, and was responsible for primary writing. EB and SL revised and approved the contents for publication. All authors contributed to the article and approved the submitted version.

## Funding

Experimental animals were funded by the USDA National Institute of Food and Agriculture, Animal Health project 1022565. Funding for the experiments came from principal investigator discretionary funds and was not connected to any specific grant.

## Conflict of interest

The authors declare that the research was conducted in the absence of any commercial or financial relationships that could be construed as a potential conflict of interest.

## Publisher’s note

All claims expressed in this article are solely those of the authors and do not necessarily represent those of their affiliated organizations, or those of the publisher, the editors and the reviewers. Any product that may be evaluated in this article, or claim that may be made by its manufacturer, is not guaranteed or endorsed by the publisher.

## Abbreviations

ADFI: Average daily feed intake
ADG: Average daily gain
BW: Body weight
CD: Cluster of differentiation
DC: Dendritic cell
FI: Feed intake
IFN: Interferon
IL: Interleukin
MHC: Major histocompatibility complex
NDV: Newcastle disease virus
PBS: Phosphate-buffered saline
PER: Proton efflux rate
pi: Post-inoculation
Rot/AA: Rotenone + antimycin A
TC: Cytotoxic T cell
TCR: T cell receptor
TH: Helper T cell
2-DG: 2-deoxy-D-glucose
