# Supplementary material for: Implementing real-time immunometabolic assays and immune cell profiling to evaluate systemic immune response variations to Eimeria challenge in three novel layer genetic lines
Source: Front Vet Sci. 2023 Apr 18;10:1179198. doi: 10.3389/fvets.2023.1179198 (PMC10153671; doi:10.3389/fvets.2023.1179198)
Supplement: Supplementary file 1 [file Data_Sheet_1.docx]

Supplementary Material

Implementing real-time immunometabolic assays and immune cell profiling to evaluate systemic immune response variations to *Eimeria* challenge in three novel layer genetic lines

K. Fries-Craft, S.J. Lamont, E.A. Bobeck^*^

*** Correspondence:** Corresponding Author: eabobeck@iastate.edu

## Supplementary Figures

**Supplemental Figure 1:** Compiled glycolytic rate assay outcomes in peripheral blood mononuclear cells isolated from Leghorn (Ghs6 and Ghs13) and Fayoumi (M5.1) inbred genetic lines at 1, 3, 7, and 10d post-inoculation (pi) with 10X Coccivac-B52 (Merck Animal Health, Kenilworth, NJ) as determined by the Agilent Glycolytic Rate Assay and Seahorse XFe96 analyzer (Santa Clara, CA). Compiled assay outcomes include average (**A**) glycolytic and (B) total proton efflux rate before addition of mitochondrial inhibitors, (**C**) compensatory glycolysis as cells work to meet metabolic needs following mitochondrial inhibition, and (**D**) residual glycolysis following inhibition by 2-deoxy-D glucose (2DG). Data represent the mean proton efflux measurement (PER; n = 5 birds/treatment) ± SEM. Bars without overlapping lettering are significantly different, *P* ≤ 0.05.
